# Passivation ability of graphene oxide demonstrated by two-different-metal solar cells

**DOI:** 10.1186/1556-276X-9-696

**Published:** 2014-12-23

**Authors:** Wen-Tzu Hsu, Zong-Sian Tsai, Liang-Chun Chen, Guan-Yu Chen, Chun-Chieh Lin, Mei-Hsin Chen, Jenn-Ming Song, Chu-Hsuan Lin

**Affiliations:** Department of Opto-Electronic Engineering, National Dong Hwa University, Hualien, Taiwan; Department of Electrical Engineering, National Dong Hwa University, Hualien, Taiwan; Department of Materials Science and Engineering, National Chung Hsing University, Taichung City, Taiwan

**Keywords:** Passivation, Solar cell, Graphene oxide, Two-different-metal, Hummers method

## Abstract

The study on graphene oxide (GO) grows rapidly in recent years. We find that graphene oxide could act as the passivation material in photovoltaic applications. Graphene oxide has been applied on Si two-different-metal solar cells. The suitable introduction of graphene oxide could result in obvious enhancement on the efficiency. The simple chemical process to deposit graphene oxide makes low thermal budget, large-area deposition, and fast production of surface passivation possible. The different procedures to incorporate graphene oxide in Si two-different-metal solar cells are compared, and 21% enhancement on the efficiency is possible with a suitable deposition method.

## Background

Energy from solar cells has been thought as the possible alternative to the traditional fuel energy. In order to compete with the traditional energy, increase on the efficiency of solar cells in a cost-effective way is important. For a solar module with an efficiency of 20%, 1% improvement on efficiency can correspond to 5% reduction in cost. Surface structures [1–3] and passivation [4–7] can be utilized to improve the efficiency. Passivation of bare Si surfaces can be easily achieved with hydrogen termination, alkylation, and so on, but the effect may deteriorate in a certain time [8]. Passivation by dielectric films, such as SiO_2_, SiN_x_, and Al_2_O_3_ could overcome the stability issue. The high-quality SiO_2_ is common oxide for surface passivation of Si solar cells. Al_2_O_3_ prepared by atomic layer deposition is also used due to its promising ability of passivation for Si, especially for the p-type Si. Since various oxide materials have been used for passivation of solar cells, we would like to investigate the effect of graphene oxide (GO) as the passivation layer. GO is broadly studied after the developing of graphene in recent years [9–11]. The above mentioned oxide passivation films are almost demonstrated in chambers, and GO deposited in chemical solution may be a much simpler method. For the photovoltaic applications, GO has been adopted in organic solar cells as the hole transport layer [12]. We will apply GO to Si solar cells with the purpose of surface passivation. The different procedures to incorporate GO in Si two-different-metal solar cells are compared. To the best of our knowledge, GO has not been utilized on the applications of solar cell passivation. The chemical solution method makes the low thermal budget, large-area deposition, and fast production possible.

## Methods

**Figure 1:**
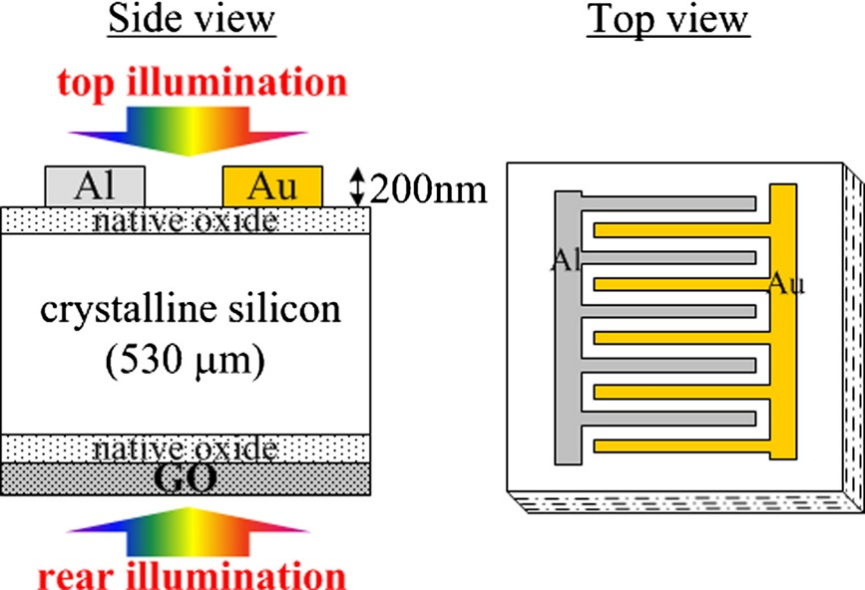
**The schematic structure of the Si two-different-metal solar cell.** The work functions of Au and Al are 5.1 and 4.18 eV, respectively, and hence, a built-in potential can be formed.

A two-different-metal structure for solar cells [13] was used in this study, because it could be fabricated easily in the laboratory and the passivation effect could be singly investigated on the side without electrodes. Figure 1 shows the schematic structure of the two-different-metal solar cell. The work functions of Au and Al are 5.1 and 4.18 eV, respectively, and hence, a built-in potential can be formed for photovoltaic application. In ref. [13], a thinned thermal SiO_2_ with high quality was inserted between the metals and Si. A critical high pressure H_2_O vapor heat treatment was also needed to obtain a satisfied interface. Since we were only interested in the effect of GO, the native oxide (SiO_2_) was kept instead of the high-quality thermal SiO_2_. We also found that if the top native oxide was removed, Au and Al might be easily shorted via the path of semiconductor. Hence, the native oxide was kept. Four kinds of cell structures (SiG_b_1, SiG_b_2, G_b_Si, and ConSi) were prepared and compared. The top sides of 1 to 30 Ω-cm p-type CZ Si substrates were evaporated by Al and Au in advance to prepare the sample ‘SiG_b_1’ and ‘SiG_b_2’. The difference between SiG_b_1 and SiG_b_2 only appeared on the procedures of GO deposition. Before the GO deposition, GO suspension should be prepared. First, graphite oxide was prepared by the modified Hummers method [14]. Subsequently, graphite oxide was added in the DI water and followed by two-step ultrasonication and centrifugation with a procedure similar to those in ref. [15, 16]. After the fist ultrasonication for 30 min, the solution was centrifuged at 4,000 r/min for 30 min. The supernatant was again ultrasonicated for 2 h followed by centrifugation at 10,000 r/min for 15 min. The ultrasonication was performed to exfoliate the GO sheets from graphite-oxide multilayer flakes. The GO sheets were hydrophilic due to their oxygen-containing groups, and the supernatant after centrifugation was dispersed with smaller and thinner flakes. The final supernatant was the GO suspension. The SiG_b_1 sample was prepared via the dip coating method. The topside-down Si substrate was immersed in the GO suspension for 40 min to coat GO sheets on the side without electrodes (rear side), and it was taken out to dry naturally to obtain the SiG_b_1 sample. To fabricate the SiG_b_2 sample, the GO suspension was only dropped on the rear surface of the Si substrate instead of the immersion of the entire substrate. This SiG_b_2 sample was dried at 70°C on the hot plate. Then, the SiG_b_2 sample was obtained. In ref. [15], the Si substrates with hydrophilic treatment in the Standard Cleaning 1 (SC1) solution with NH_4_OH:H_2_O_2_:H_2_O = 1:2:8 before GO deposition could own a dense coverage of GO. We would like to prepare a similar sample with SC1 treatment. However, we found that the electrodes of the cells would exfoliate when immersing in the SC1 solution. Hence, we prepared a sample named as ‘G_b_Si’, where the Si substrate was SC1 (hydrophilicly) treated in advance. Dip coating and drying of GO suspension were then performed, and electrodes were evaporated finally. A similar two-metal structure without the GO deposition, named as ‘ConSi’, was also fabricated for comparison.

## Results and discussion

**Figure 2:**
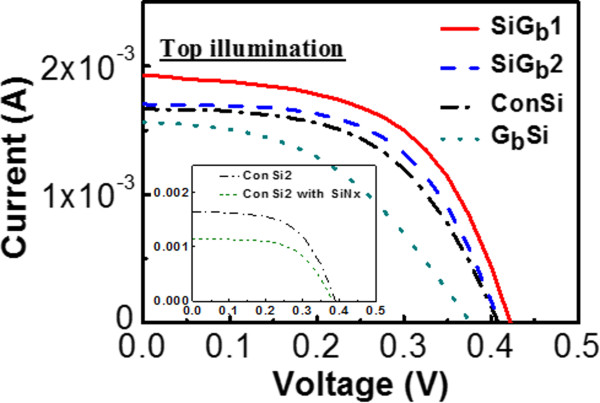
**The current versus voltage characteristics with AM 1.5 G illumination from the top side.** The device area is 0.72 cm^2^. The inset shows that the deposited SiNx film results in a poor performance.

**Figure 3:**
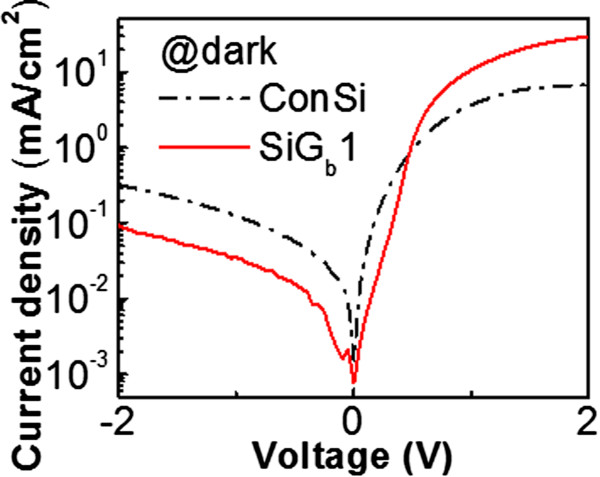
**The dark current density versus voltage characteristics of SiG**_**b**_ **1 and ConSi.** The smaller dark reverse (negative bias) current density of the SiG_b_1 cell contributes to its higher *V*_OC_ as compared to ConSi.

The current versus voltage (*IV*) characteristics of the samples with AM 1.5 G illumination from the top side (the side with electrodes) are shown in Figure 2. When light is irradiated from the top surface, the short-circuit current (*I*_SC_) of the SiG_b_1, SiG_b_2, ConSi, and G_b_Si cells are 1.930, 1.703, 1.603, and 1.563 mA, respectively. The open-circuit voltage (*V*_OC_) are 0.423, 0.409, 0.408, and 0.376 V, respectively. Both SiG_b_1 and SiG_b_2 show better performance as compared with the reference ConSi cell. It proves that GO indeed owns the ability to passivate the Si solar cells. It should be mentioned that in the current stage, the solar cell is not optimized on the efficiency, and hence, the efficiency is not good enough. Hence, we only focus on *I*_SC_ and *V*_OC_ in order to indicate the influence contributed by GO. Because the Au and Al should be evaporated by shadow masks in different runs, the alignment between runs limits the pattern. The pattern could not be optimized just considering carrier generation and collection. The device area is 0.72 cm^2^, but the effective area without electrode shielding is only 0.19 cm^2^. Hence, the best GO (SiG_b_1) and control cells (ConSi) can only own efficiencies of 0.63% and 0.52%, respectively. Although the absolute value of efficiency is low due to the un-optimized cell structure, the results indicate that the GO introduction can contribute up to 21% enhancement on the efficiency. The dark current density versus voltage characteristics of SiG_b_1 and ConSi are also shown in Figure 3. The smaller dark reverse (negative bias) current density of the SiG_b_1 cell contributes to its higher *V*_OC_ as compared to ConSi [17].

**Figure 4:**
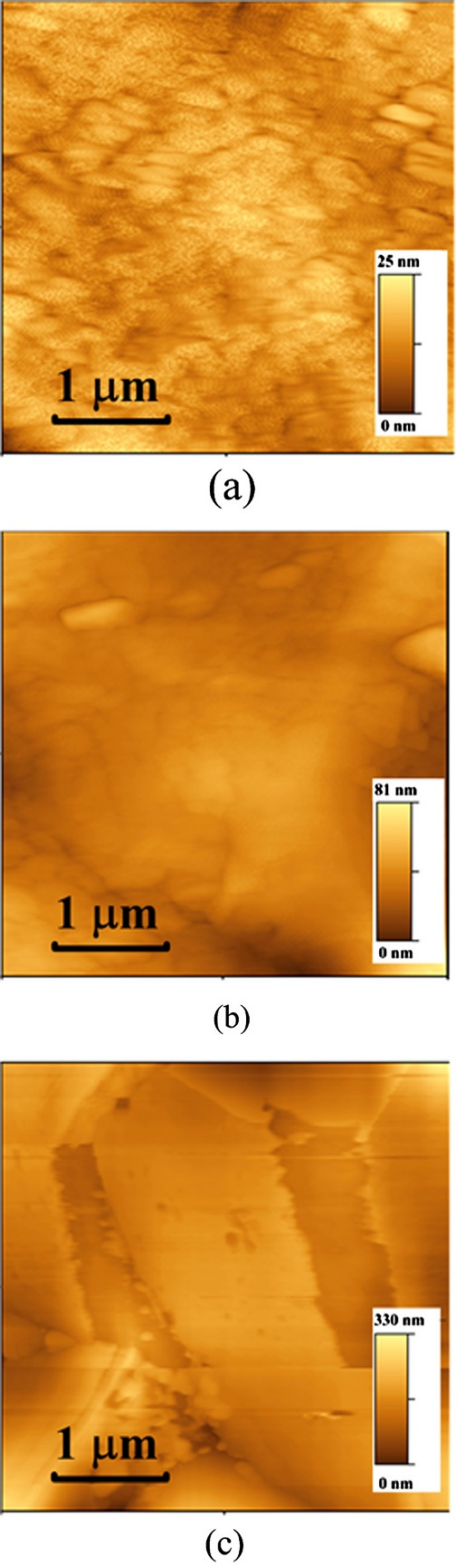
**The AFM images of (a) SiG**_**b**_ **1, (b) SiG**_**b**_ **2, and (c) G**_**b**_ **Si samples.** For the sample of SiG_b_1, the deposited GO film was approximately 20 nm in thickness. On the other hand, the GO film on G_b_Si is much thicker due to the extra SC1 treatment before GO deposition.

The other GO sample, G_b_Si, shows a worse performance than the ConSi. In order to find the reason for degradation of G_b_Si, the atomic force microscope (AFM) images of samples with GO deposition were measured (Figure 4). The AFM images show that the SC1 treatment resulted in a much thick GO film on the rear side of Si of G_b_Si (Figure 4c) as compared with those of SiG_b_1 (Figure 4a) and SiG_b_2 (Figure 4b). The highly hydrophilic surface of G_b_Si may also attract GO flakes on the top side (sides with electrodes). The GO flakes between Si and electrodes would prevent the current conduction, which results in the poor performance of GbSi as compared to the ConSi.

The passivation effect of GO is supposed due to the field effect passivation contributed by the negative fixed charge in GO as verified in ref. [15]. In ref. [15], the capacitance verse voltage relation of GO and control metal-insulator-semiconductor (MIS) capacitors were compared. The curves of MIS capacitors with GO coated shifted to the positive-bias direction, and it meant that extra negative charge existed in GO. Such a dielectric GO film with negative fixed charge could be used to passivate solar cells, especially for the p-side. For our two-different-metal solar cells, GO can be coated on the rear side of the p-type Si substrate. Without GO being coated, many of the photo-generated electron-hole pairs may easily recombine at the rear surface due to the termination of the periodic Si structure. With GO coated, minority carriers (electrons) can be repelled from the surface. Since recombination should only occur between an electron and a hole, the repulsion of electron from the surface could contribute to the decrease of recombination. More electrons can be collected by the Al electrode successfully, and hence, more holes can be collected by the Au electrode. That is why SiG_b_1 and SiG_b_2 show the better performance as compared with the ConSi.

Silicon nitride (SiN_x_) is a common passivation film for solar cells. We have also prepared a two-different-metal solar cell with SiN_x_ on the rear side for comparison. First, we demonstrated another control two-different-metal solar cell, ConSi2, and its *IV* characteristic under AM 1.5 G illumination was obtained as shown in the inset of Figure 2. Then, the native oxide on the rear side of ConSi2 was removed by buffered oxide etch (BOE). SiN_x_ was subsequently deposited on the rear side by the sputter. Its *IV* characteristic is also shown in the inset of Figure 2 as the curve of ‘ConSi2 with SiN_x_’. It can be found that the performance of ‘ConSi2 with SiN_x_’ is even worse than ConSi2. One reason for the degradation may be due to the un-optimized facility for passivation. The sputter SiN_x_ might have poor quality as compared with the commercial PECVD SiN_x_. The other reason is that SiN_x_ with positive fixed charge is more suitable for passivation of n-Si substrates instead of p-Si substrates in our case [18].

Because the best GO cell, SiG_b_1, has been immersed in the GO suspension for 40 min, it may be suspected that the performance enhancement is due to the more oxidation in water (in GO suspension) but not GO deposition. We prepared two extra control samples. One was immersed in DI water, and the other was rear-side down floating on the water surface of GO suspension to have the similar immersion condition but avoid GO deposition. These two control samples after immersion did not show better cell performance than the results before immersion (not shown here), indicating that the improvement was indeed only due to the GO passivation on the surface.

**Figure 5:**
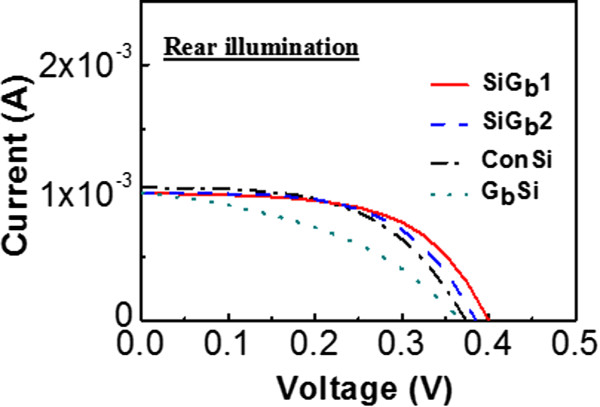
**Current versus voltage characteristics with AM 1.5 G illumination from the rear side.** With rear illumination, *I*_SC_ of the SiG_b_1, SiG_b_2, ConSi, and G_b_Si cells are 1.000, 0.999, 1.040, and 1.000 mA, and *V*_OC_ are 0.400, 0.383, 0.374, and 0.370 V, respectively. GO samples own smaller *J*_SC_ as compared to ConSi because partial input light will be absorbed by GO on the rear side.

**Figure 6:**
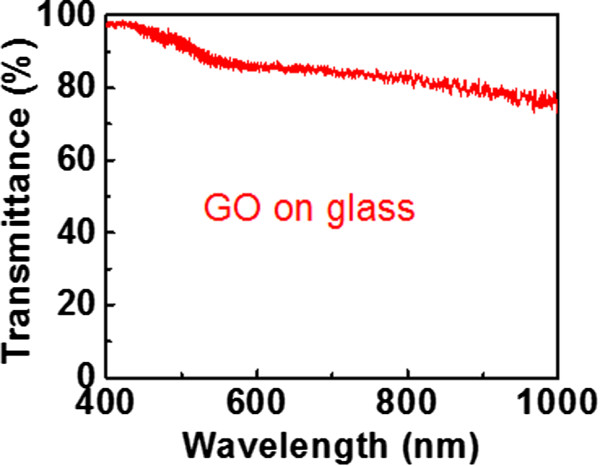
**The transmittance of the glass with a GO film.** The deposition condition of this GO film on glass is similar to that of SiG_b_1. The reference background in this FTIR measurement is air instead of glass to avoid the large fluctuation after dividing by background.

In ref. [13], the light illumination from the rear surface results in a larger short-circuit current density (*J*_sc_) than that from the top surface, which is due to the avoidance of electrode shading. However, the *I*_SC_ of our control cell (ConSi) with rear illumination (Figure 5) is smaller than top illumination (Figure 2), since the rear side of our control cell is not coated by the 100-nm-thick oxide (performed in ref. [13]). Furthermore, the *I*_SC_ of all GO samples is even smaller than that of ConSi. It is suspected that some of incident light is absorbed by GO. The transmittance spectrum of GO on glass has been measured by Fourier transform infrared spectroscopy (FTIR) (Figure 6). In Figure 6, with an incident wavelength of 550 nm, the transmittance of GO on glass is only 87%. The deposition of GO reduces the amount of light entering the Si, and hence the *I*_SC_ of the GO samples is smaller than that of the control sample for the rear-side illumination case. Although *I*_SC_ of SiG_b_1 and SiG_b_2 can not be superior than ConSi, *V*_OC_ of both samples can still be superior than ConSi. The lower recombination contributed by GO corresponds to a smaller reverse current, which results in a larger *V*_OC_ as mentioned above.

## Conclusions

GO is first-time proven to have the ability to enhance the performance of a solar cell by surface passivation due to its negative fixed charge. GO provides the potential on low-cost and large-area passivation. In the current stage, the simple two-different-metal structure is adopted as the beginning. Further optimization on deposition conditions and light transmission is deserved. More efforts should be made to incorporate the benefit of GO in commercial Si pn solar cells.
